# Early detection of kidney impairment in school-aged children born very preterm: a parallel use of traditional and modern biomarkers

**DOI:** 10.1007/s00467-025-06876-1

**Published:** 2025-07-07

**Authors:** Vaia Dokousli, Nikolaos Gkiourtzis, Anastasia Stoimeni, Despoina Samourkasidou, Kali Makedou, Christos Tsakalidis, George Koliakos, Despoina Tramma

**Affiliations:** 1 4th Department of Pediatrics, “G. Papageorgiou” General Hospital, School of Medicine, Faculty of Health Sciences, Aristotle University of Thessaloniki, Thessaloniki, Greece; 2 Laboratory of Biochemistry, AHEPA University Hospital, School of Medicine, Faculty of Health Sciences, Aristotle University of Thessaloniki, Thessaloniki, Greece; 3 2nd Neonatal Department and Neonatal Intensive Care Unit, “G. Papageorgiou” General Hospital, School of Medicine, Faculty of Health Sciences, Aristotle University of Thessaloniki, Thessaloniki, Greece; 4 Laboratory of Biological Chemistry, School of Medicine, Faculty of Health Sciences, Aristotle University of Thessaloniki, Thessaloniki, Greece

**Keywords:** Prematurity, Preterm children, Biomarkers, Cystatin C, Symmetric dimethylarginine, Kidney function

## Abstract

**Background:**

Prematurity has been linked to kidney dysfunction from infancy through adulthood. Children born very preterm are at particular risk due to interrupted nephrogenesis. However, early detection remains challenging, and a uniform monitoring strategy is lacking.

**Methods:**

This cross-sectional study involved school-aged (6–16 years) children born at ≤ 32 weeks of gestation, with no history of small for gestational age (SGA). They were further stratified by birth weight (BW): low, very low, and extremely low (LBW, VLBW, ELBW) categories. Age- and sex-matched full-term children served as controls. Anthropometry, blood pressure (BP), and kidney function were assessed, using traditional (urea; creatinine, Cr; β2-microglobulin, B2M; albuminuria) and modern biomarkers (cystatin C, CysC; symmetric dimethylarginine, SDMA). Estimated glomerular filtration rate (eGFR) based on Cr and Cr-CysC was also calculated. Statistical analysis was performed using R (version 4.3.2), with significance set at *p* < 0.05.

**Results:**

Eighty-one children were included: 43 preterm (77% from multiple pregnancies) and 38 controls. Compared to controls, preterm participants had higher serum cystatin C (*p* < 0.001) and lower Cr-CysC-eGFR (*p* < 0.001). They also had higher serum urea (*p* = 0.002), but all individual values were within the normal range. No differences were observed in BP, serum Cr, Cr-eGFR, or albuminuria. ELBW children had lower body mass index (BMI) (*p* = 0.048) and higher B2M (*p* = 0.046) than LBW peers.

**Conclusions:**

School-aged children born very preterm may already exhibit subtle signs of kidney dysfunction, with ELBW children showing greater metabolic and renal strain. Cystatin C and Cr-CysC-eGFR appear promising biomarkers for early detection of kidney alterations in this high-risk population.

**Graphical abstract:**

**Figure:**
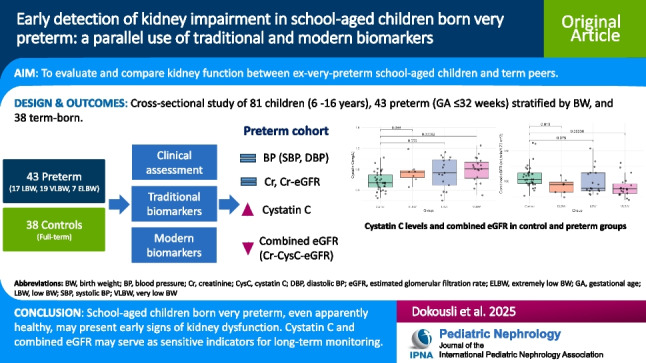
A higher resolution version of the Graphical abstract is available as Supplementary information

**Supplementary Information:**

The online version contains supplementary material available at 10.1007/s00467-025-06876-1.

## Introduction

Prematurity, defined as birth before the gestational age (GA) of 37 weeks, remains a major global concern, affecting approximately 11% of live births worldwide. Advances in neonatal care have significantly improved survival rates of preterm infants, raising plausible questions about their long-term health outcomes, especially for those born at the threshold of viability [1]. These concerns align with the Developmental Origins of Health and Disease (DOHaD) hypothesis, which postulates that adverse in utero or early life environmental exposures can predispose individuals to chronic diseases including hypertension, diabetes, and kidney disease. Prematurity per se, or through its association with adverse exposures, may trigger developmental programming changes [2, 3].

Preterm birth, especially before 32 weeks of gestation, interrupts nephrogenesis, which normally continues until approximately 36 weeks, resulting in reduced nephron endowment. Notably, over 60% of nephrons are formed during the third trimester of pregnancy. According to Brenner’s hypothesis, fewer nephrons at birth induce compensatory hyperfiltration, hypertrophy, and progressive nephron loss, increasing the risk of chronic kidney disease (CKD) and hypertension [4]. Additionally, up to 30% of neonates in neonatal intensive care units (NICUs) experience acute kidney injury (AKI), potentially compounding nephron deficit and long-term dysfunction [5]. Definitions of prematurity and related perinatal conditions associated with nephron deficit are summarized in Table 1 [1, 6].

**Table 1:** Definitions of prematurity and related perinatal conditions^†^

Terminology	Definition	Time of diagnosis	Notes
Prematurity or preterm birth	GA < 37 weeks	Postnatal	Includes all births < 37 weeks
Late preterm	34 ≤ GA < 37 weeks	Postnatal	—
Moderately preterm	32 ≤ GA < 34 weeks		
Very preterm	28 ≤ GA < 32 weeks		
Extremely preterm	GA < 28 weeks		
Low birth weight (LBW)	BW < 2500 g	Postnatal	May include preterm or growth-restricted infants
Very low birth weight (VLBW)	1000 ≤ BW < 1500 g	Postnatal	—
Extremely low birth weight (ELBW)	BW < 1000 g		
Small for gestational age (SGA)	BW < 10th percentile for GA and gender	Postnatal	May include constitutionally small or growth-restricted infants
Intrauterine or fetal growth restriction (IUGR/FGR)	Failure to reach growth potential in utero	Prenatal	Diagnosed by fetal ultrasound biometrics and Doppler

*BW* birth weight, *GA* gestational age

^†^Definitions based on Deffrennes et al. (2024) and Terstappen and Lely (2020) [1, 6]

Despite mounting evidence, the precise mechanisms and timing of kidney alterations during childhood and adolescence in preterm populations remain unclear [1]. Observational studies comparing kidney health outcomes between preterm and full-term children have yielded inconsistent results [7–13], while monitoring guidelines remain unstandardized [1]. Furthermore, routine biomarkers like serum creatinine (Cr) and Cr-based estimated glomerular filtration rate (Cr-eGFR), may miss early kidney impairment, while alternative biomarker candidates remain underexplored in this context [14, 15].

The present study aimed to investigate kidney function in apparently healthy, school-aged children (6 to 16 years) born very preterm (≤ 32 weeks GA), compared to full-term peers. By assessing a range of clinical and laboratory parameters, incorporating both traditional (Cr; urea; urinary albumin-to-creatinine ratio, uACR; β2-microglobulin, B2M) and modern biomarkers (cystatin C, CysC; symmetric dimethylarginine, SDMA), we sought to provide further insight into early and subclinical alterations associated with this degree of prematurity. Cystatin C, a low-molecular-weight protein freely filtered by the glomerulus without tubular secretion, was selected as a well-established, sensitive, and increasingly accessible marker for early kidney dysfunction [16, 17]. B2M was chosen as a biomarker relevant to both glomerular and tubular function [18, 19]. SDMA was employed as a promising marker of early kidney impairment and endothelial dysfunction, independent of muscle mass, and stable across pediatric age groups [20, 21]. Given the limited data on B2M and SDMA in contexts like our study, their inclusion aimed to address existing knowledge gaps [9, 10, 22].

## Materials and methods

###  Study design

This cross-sectional study was conducted at the pediatric nephrology outpatient clinic of our institution between October 2021 and May 2024. Eligible participants were apparently healthy children monitored for their kidney function as part of a preventive follow-up due to prematurity. Inclusion criteria were age 6–16 years at study entry; GA ≤ 32 weeks; birth weight (BW) not classified as small for gestational age (SGA); and absence of severe neurodevelopmental delay. Concurrently, healthy children born full-term (GA ≥ 37 weeks), matched for age and sex, were recruited as controls from the general pediatrics outpatient clinic, which, despite being part of a tertiary hospital, also provides preventive services and health certifications for routine pediatric care. Exclusion criteria for both groups were congenital anomalies/genetic disorders; congenital anomalies of the kidney and urinary tract (CAKUT); personal or family history of kidney disease; history of recurrent urinary tract infections (≥ 2 episodes, per parent/guardian report). Participants were consecutively enrolled during scheduled clinic visits until the required sample size was reached, ensuring a systematic selection process.

The target sample size was predetermined based on the study by Kwinta et al. [7], using serum cystatin C as the primary outcome. A minimum of 62 participants (31 per group, 1:1 ratio) was calculated to achieve 80% statistical power at a significant level of *α* = 0.05 with the Sample Size Calculator program.

All participants were enrolled after obtaining written informed consent from their parents or legal guardians. The research protocol has been registered in OSF (https://osf.io/zjcr9) and was approved by the Local Bioethics and Ethics Committee of the School of Medicine (Approval No. 1499/19.10.2021).

### Data collection and clinical assessment

Neonatal data were extracted from NICU records and parental interviews. Collected variables included (i) pregnancy data: gestational diabetes, pregnancy-induced hypertension or preeclampsia, antenatal steroids, prolonged prelabor rupture of membranes (> 24 h), gestation type, zygosity, twin-to-twin transfusion syndrome (TTTS), and fetal growth restriction (FGR); (ii) perinatal data: mode of delivery, sex, APGAR score, BW, GA, extreme prematurity (EPT), multiple birth (twins/triplets) and zygosity status (monozygotic/dizygotic/trizygotic); (iii) NICU stay details: duration of stay, mechanical ventilation, neonatal co-morbidities (acute respiratory distress syndrome-ARDS, bronchopulmonary dysplasia-BPD, necrotizing enterocolitis-NEC, intraventricular hemorrhage-IVH, patent ductus arteriosus-PDA, sepsis, AKI, nephrocalcinosis), umbilical catheterization, exposure to nephrotoxic antibiotics (gentamycin, amikacin, vancomycin, teicoplanin), and other medications with known kidney impact (postnatal steroids, diuretics, non-steroidal anti-inflammatory drugs-NSAIDs for PDA closure). SGA was defined as BW below the 10th percentile (*z*-score ≈ − 1.28) for the GA and sex, based on Fenton growth charts [23]. Neonatal AKI was defined according to modified KDIGO criteria: increase in serum Cr by ≥ 0.3 mg/dL (26.5 µmol/L) or ≥ 50% from the previous lowest value, and/or urine output < 1 mL/kg/h for 24 h, regardless of postnatal age [5]. For the full-term group, perinatal data were retrieved from the individual child’s health record.

Participants underwent a single, system-based clinical examination during their outpatient visit. Body weight and height were measured using a calibrated digital scale (accuracy of 0.1 kg) and a fixed wall-mounted stadiometer (accuracy of 0.1 cm), respectively, with children barefoot and in light clothing. Body mass index (BMI) was calculated using the formula: BMI = weight (kg) ÷ [height (m)]^2^. Overweight was defined as a BMI at or above the 85th and below the 97th percentile, and obesity as a BMI above the 97th percentile for age and sex, according to WHO growth charts [24].

Waist circumference (WC) was measured at the midpoint between the iliac crest and lowest rib using a flexible tape (accuracy of 0.1 cm), on a relaxed abdomen. Central adiposity was defined as WC above the 90th percentile for age and sex, based on Greek reference data [25].

After 10 min of rest, blood pressure (BP) was measured on the right arm using an appropriately sized cuff and validated oscillometric device (Microlife BP B1 Classic), with the limb supported at heart level. Three readings were taken at 3-min intervals and averaged for analysis. BP ≥ 95th percentile (systolic–SBP or diastolic–DP), based on American Academy of Pediatrics (AAP) 2017 guidelines, was considered potentially abnormal and flagged for follow-up [26].

### Kidney function studies

Following detailed medical history and clinical examination, fasting blood and first-morning urine samples were obtained from all participants, who were adequately hydrated. Blood samples were collected via antecubital venipuncture under standardized conditions. Hemoglobin (Hb), serum Cr, urea, B2M and parathormone (PTH), as well as urine albumin, calcium (Ca) and Cr were measured in standard automated hospital analyzers. Albuminuria was defined as a urinary albumin-to-creatinine ratio (uACR) ≥ 30 mg/g [17], and normal urinary (U) Ca excretion as U_Ca_/U_Cr_ ratio < 0.22. Serum samples for cystatin C and SDMA were promptly frozen at − 30 °C and subsequently analyzed in a single batch using enzyme-linked immunosorbent assay (ELISA) kits: sandwich ELISA for CysC (Boster Biological Technology, EK0678) and competitive ELISA for SDMA (Immundiagnostik AG, Germany). Both assays demonstrated acceptable intra- and inter-assay variability (CV < 10%) [27]. Estimated GFR (eGFR) was calculated using the updated bedside Schwartz equation for Cr and the combined CKiD Cr-CysC-eGFR formula as recommended by the National Kidney Foundation [28].

### Statistical analysis

Statistical analysis was performed using R software (version 4.3.2) [29]. Continuous variables with normal distribution are presented as mean with standard deviation [mean (± SD)], while non-normally distributed variables are presented as median with interquartile range [median (Q1, Q3)]. Group comparisons were conducted using independent-sample *t*-tests for normally distributed variables and the Mann–Whitney *U* test for skewed data. Categorical variables were compared using the chi-square (*χ*^2^) test or Fisher’s exact test, as appropriate. A *p*-value < 0.05 was considered statistically significant.

## Results

### Study population

**Fig. 1:**
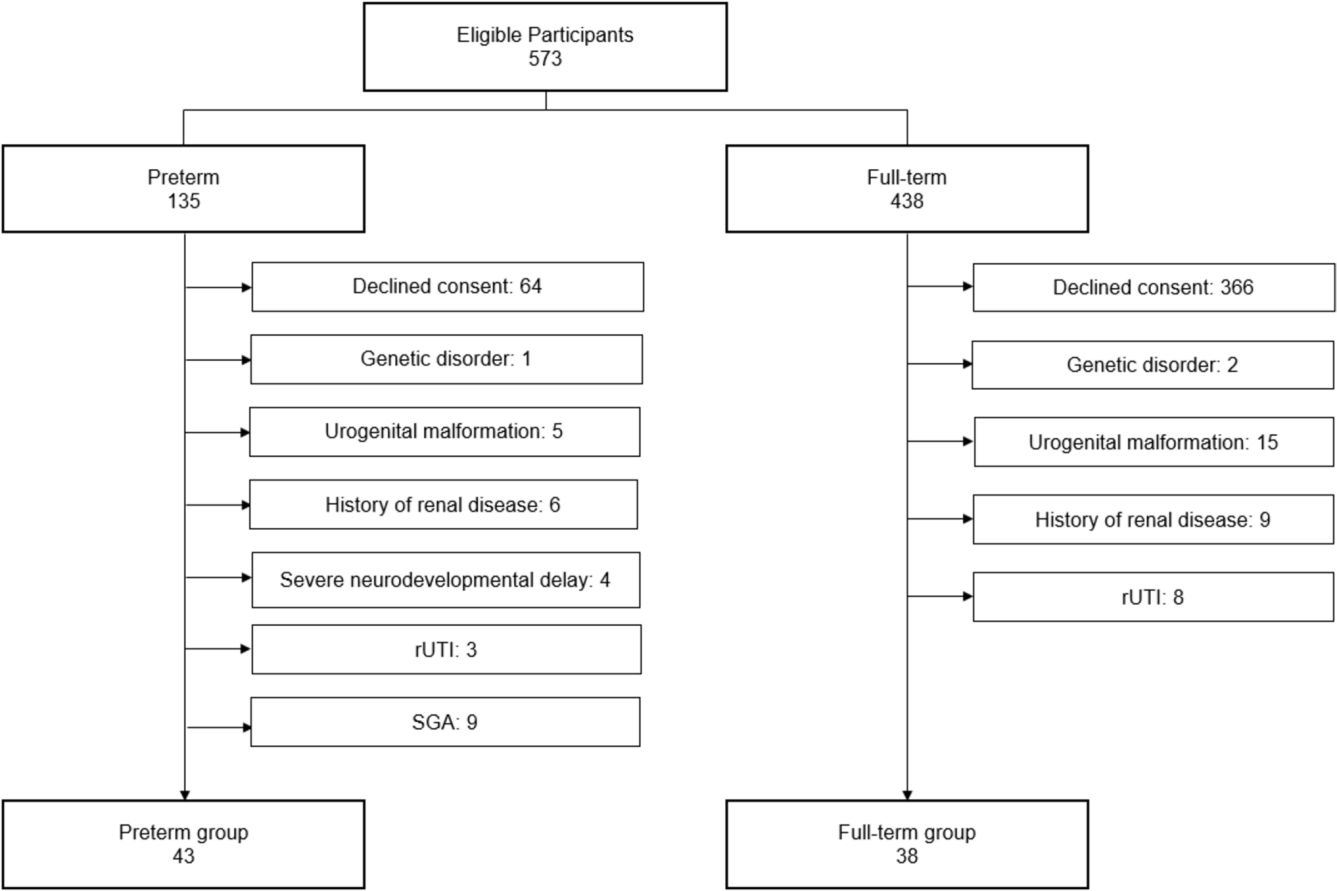
Flow chart of the cross-sectional study. *rUTI* recurrent urinary tract infection, *SGA* small for gestational age

A total of 81 participants were enrolled, comprising 43 ex-very-preterm individuals (preterm group) and 38 age- and sex-matched controls (reference group). BW and GA did not differ between participants and those who declined participation. The recruitment flowchart is shown in Fig. 1. The mean age of the participants was 10.43 (± 2.55) years, with 49% being female.

The 43 preterm participants were born from 27 pregnancies, 17 of which were multiple gestations (13 twins and 4 triplets), yielding 33 out of the 43 preterm individuals (77%). Pregnancy, perinatal, and NICU data of the preterm participants, both overall and by BW categories are summarized in Table 2. Among them, 17 (40%) were classified as low BW (LBW), 19 (44%) as very low BW (VLBW), and 7 (16%) as extremely low BW (ELBW). The mean BW was 1388.72 (± 358.70) grams, and the median GA was 31.00 [29.14, 31.86] weeks. TTTS was documented in one pair of monozygotic male twins; the donor twin was excluded due to SGA status. Four preterm participants had FGR (maximum duration: 1 week – one from a monozygotic twin pair, one from a dizygotic twin pair, and two from singleton pregnancies.

**Table 2:** Pregnancy, perinatal, and NICU data of preterm participants by birth weight category

	PT-LBW *n* = 17	PT-VLBW *n* = 19	PT-ELBW *n* = 7	PT-ALL *n* = 43
Pregnancy data				
Gestational diabetes	4 (23.5%)	1 (5.3%)	0	5 (11.6%)
Pregnancy-induced HT or preeclampsia	4 (23.5%)	1 (5.3%)	3 (42.9%)	8 (18.6%)
Antenatal steroids	14 (82.4%)	16 (84.2%)	5 (71.4%)	35 (81.4%)
Prolonged prelabor rupture of membranes (> 24 h)	1 (5.9%)	4 (21.1%)	5 (71.4%)	10 (23.3%)
FGR	0	1 (5.3%)	3 (42.9%)	4 (9.3%)
TTTS	0	1 (5.3%)	0	1 (2.3%)
Perinatal data				
Cesarean section	17 (100%)	19 (100%)	6 (85.7%)	42 (97.7%)
Female sex	9 (52.9%)	10 (52.6%)	4 (57.1%)	23 (53.5%)
Apgar 1 min	8.00 (7.00, 8.00)	8.00 (7.00, 8.00)	6.00 (5.50, 7.00)	7.50 (7.00, 8.00)
Apgar 5 min	9.00 (8.00, 9.00)	8.00 (7.25, 9.00)	8.00 (7.25, 8.00)	8.00 (8.00, 9.00)
Birth weight (g)	1747.06 (± 183.47)	1275.00 (± 97.60)	827.14 (± 98.10)	1388.72 (± 358.70)
EPT	0	1 (5.3%)	6 (85.7%)	7 (16.3%)
Gestational age (weeks)	31.86 (31.57, 32.00)	29.71 (29.43, 31.43)	25.57 (25.29, 27.71)	31.00 (29.14, 31.86)
Children born from multiple pregnancies	17 (100%)	14 (73.7%)	2 (28.6%)	33 (76.7%)
Monozygotic	4	4	2	10
Dizygotic	11	9	0	20
Trizygotic	2	1	0	3
NICU stay overview				
Length of NICU hospitalization (days)	29.00 (23.00, 31.00)	35.50 (34.00, 57.50)	95.00 (71.50, 98.50)	34.50 (29.25, 58.50)
Mechanical ventilation	7 (41.2%)	11 (57.9%)	6 (85.7%)	24 (55.8%)
Length of mechanical ventilation (days)	1.00 (0.75, 2.50)	2.00 (1.50, 5.00)	24.50 (11.25, 28.75)	2.50 (1.00, 7.00)
ARDS	10 (58.8%)	11 (57.9%)	6 (85.7%)	27 (62.8%)
Bronchopulmonary dysplasia	0	4 (21.1%)	6 (85.7%)	10 (23.3%)
Necrotizing enterocolitis	0	0	1	1 (2.3%)
Intraventricular hemorrhage	1 (5.9%)	2 (10.5%)	5 (71.4%)	8 (18.6%)
PDA	0	3 (15.8%)	3 (42.9%)	6 (14.0%)
PDA ligation	0	0	3	3
NSAID treatment for PDA^†^	0	3	3	6
Umbilical catheterization	7 (41.2%)	15 (78.9%)	7 (100%)	29 (67.4%)
Sepsis	2 (11.8%)	5 (26.3%)	5 (71.4%)	12 (27.9%)
AKI	0	0	0	0
Nephrocalcinosis	0	0	2 (28.6%)	2 (4.7%)
Gentamycin treatment	9 (52.9%)	14 (73.7%)	6 (85.7%)	29 (67.4%)
Duration of gentamycin treatment (days)	5.00 (3.00, 5.00)	5.00 (4.00, 7.50)	8.00 (7.00, 11.25)	5.00 (4.00, 8.00)
Amikacin treatment	4 (23.5%)	10 (52.6%)	7 (100%)	21 (48.8%)
Duration of amikacin treatment (days)	7.00 (6.25, 7.25)	4.00 (3.00, 7.00)	10.00 (6.00, 13.00)	7.00 (4.00, 8.00)
Vancomycin treatment	7 (41.2%)	14 (73.7%)	7 (100%)	28 (65.1%)
Duration of vancomycin treatment (days)	7.00 (5.50, 8.00)	7.00 (4.50, 10.75)	18.00 (10.50–25.00)	7.00 (4.00, 14.75)
Teicoplanin treatment	1 (5.9%)	4 (21.1%)	5 (71.4%)	9 (20.9%)
Duration of teicoplanin treatment (days)	10.00	8.00 (4.00, 8.00)	10.00 (9.00, 11.00)	9.00 (8.00, 10.00)
Postnatal steroids	1 (5.9%)	4 (21.1%)	6 (85.7%)	11 (25.6%)
Diuretics	1 (5.9%)	7 (36.8%)	7 (100%)	15 (34.9%)

*AKI* acute kidney injury, *ARDS* acute respiratory distress syndrome, *BW* birth weight, *ELBW* extremely low BW, *EPT* extremely preterm, *FGR* fetal growth restriction, *HT* hypertension, *LBW* low BW, *NICU* neonatal intensive care unit, *NSAID* non-steroidal anti-inflammatory drugs, *PDA* patent ductus arteriosus, *PT* preterm, *SGA* small for gestational age, *TTTS* twin-to-twin transfusion syndrome, *VLBW* very low BW

Data are presented as the number of participants, *n*; the number of participants with the percentage in parentheses, *n* (%); the median with interquartile range, median (Q1, Q3); mean with standard deviation, mean (± SD), as appropriate

^†^All NSAID-treated infants received either ibuprofen or indomethacin for pharmacologic closure of PDA

A trend of increasing morbidity was observed with decreasing BW and GA. Sepsis occurred in 28% of all cases and in 71% of those with ELBW. Mechanical ventilation was required in 56% of neonates, with a median duration of 2.5 days, but it was significantly prolonged in ELBW infants (24.5 days). This subgroup also exhibited the highest rates of other major complications, including ARDS (86%), intraventricular hemorrhage (71%), and bronchopulmonary dysplasia (86%).

No cases of AKI were documented. Nephrocalcinosis was reported in a pair of monozygotic twins in the ELBW subgroup. PDA was diagnosed in six preterm neonates (14%), all treated with NSAIDs (indomethacin or ibuprofen) and three required surgical ligation.

Key demographic, clinical, and laboratory parameters, along with the results of comparative analyses, are presented in Tables 3 and 4, which summarize comparisons across the different study groups and subgroups.

**Table 3:** Characteristics of preterm and control groups with statistical comparisons

Variable	Preterm group (*n* = 43)	Controls (*n* = 38)	*p*-value
Demographics			
Age at the time of assessment (years)	11.05 (8.80, 12.07)	10.20 (± 2.32)	0.25
Sex (male/female)	20/23	21/17	0.57
Male (%)	20 (46.5%)	21 (55.3%)	NA^**#**^
Perinatal data			
Gestational age (weeks)	31.00 (29.14, 31.86)	38.66 (± 1.15)	** < 0.001**
Birth weight (grams)	1388.72 (± 358.70)	3180.54 (± 590.56)	** < 0.001**
Birth length (cm)	39.00 (± 4.21)	50.00 (49.00, 51.00)	** < 0.001**
Clinical assessment			
Weight (kg)	36.10 (29.40, 45.50)	36.20 (29.20, 51.50)	0.57
Height (cm)	143.74 (± 17.16)	143.57 (± 16.14)	0.96
BMI (kg/m^2^)	17.68 (15.37, 19.78)	17.90 (16.24, 21.93)	0.20
SBP (mmHg)	113.99 (± 11.71)	114.25 (± 9.51)	0.69
DBP (mmHg)	68.46 (± 9.48)	68.27 (± 8.14)	0.93
WC (cm)	64.50 (55.35, 70.55)	66.54 (± 10.08)	0.23
Common laboratory parameters^†^			
Hb (g/dL)	13.66 (± 0.97)	13.31 (± 1.07)	0.30
Serum creatinine (mg/dL)	0.59 (± 0.09)	0.57 (± 0.11)	0.53
Serum urea (mg/dL)	27.00 (25.00, 31.00)	23.00 (20.00, 28.00)	**0.002**
Serum B2M (mg/L)	1.40 (± 0.22)	1.41 (± 0.23)	0.82
PTH (pg/mL)	39.50 (29.30, 58.50)	41.90 (31.20, 52.70)	0.80
Cr-eGFR (mL/min/1.73 m^2^)	99.66 (96.37, 108.30)	105.71 (± 14.87)	0.18
Urinary Ca/Cr	0.09 (0.04, 0.14)	0.06 (0.04, 0.12)	0.50
Albuminuria (uACR ≥ 30 mg/g)	***n* = 42**	***n* = 34**	
	4 (9.5%)	3 (8.8%)	1
Modern parameters^‡^			
	***n* = 43**	***n* = 34**	
Serum cystatin C (mg/L)	0.76 (± 0.26)	0.57 (± 0.18)	** < 0.001**
Combined Cr-CysC-eGFR (mL/min/1.73 m^2^)	87.64 (81.90, 101.31)	102.86 (96.01, 113.57)	** < 0.001**
Serum SDMA (μmol/L)	0.81 (0.68, 0.87)	0.80 (0.73, 0.87)	0.89

*B2M* β2-microglobulin, *BMI* body mass index, *Ca* calcium, *Cr* creatinine, *CysC* cystatin C, *DBP* diastolic blood pressure, *eGFR* estimated glomerular filtration rate, *Hb* hemoglobin, *PTH* parathormone, *SDMA* symmetric dimethylarginine, *SBP* systolic blood pressure, *uACR* urinary albumin to creatine ratio, *WC* waist circumference

Data are presented as the number of participants, *n*; the number of participants with the percentage in parentheses, *n* (%); the median with interquartile range, median (Q1, Q3); the mean with standard deviation, mean (± SD), as appropriate

^#^*NA*, not applicable; no statistical comparison was performed for descriptive percentages

^†^Includes traditional biomarkers (as defined in the text), plus Cr-eGFR

^‡^Includes modern biomarkers (as defined in the text), plus Cr-CysC-eGFR

**Table 4:** Characteristics of preterm subgroups and controls with statistical comparisons

Variable	Preterm group (*n* = 43)			Controls (*n* = 38)	*p*-value
	LBW (*n* = 17)	VLBW (*n* = 19)	ELBW (*n* = 7)		
Perinatal data					
Birth weight (grams)	1747.06 (± 183.47)	1275.00 (± 97.60)	810.00 (730.00, 920.00)	3180.54 (± 590.56)	^**1**^** < 0.001** ^**2**^** < 0.001** ^**3**^** < 0.001 ** ^**4**^** < 0.001**
Birth length (cm)	41.56 (± 3.33)	39.00 (36.00, 41.00)	34.00 (32.50, 35.00)	50.00 (49.00, 51.00)	^**1**^** < 0.001** ^**2**^** < 0.001** ^**3**^** < 0.001** ^**4**^** < 0.001**
Clinical assessment					
Weight (kg)	41.50 (35.30, 54.20)	35.44 (± 14.67)	33.50 (23.60, 36.10)	36.20 (29.20, 51.50)	^1^ 0.26 ^2^ 0.20 ^3^ 0.15 ^**4**^**0.04**
Height (cm)	150.64 (± 12.77)	139.63 (± 20.67)	142.90 (127.50, 147.20)	143.57 (± 16.14)	^1^ 0.16 ^2^ 0.46 ^3^ 0.36 ^**4**^**0.03**
BMI (kg/m^2^)	19.77 (17.38, 21.44)	17.39 (± 2.77)	16.27 (13.89, 17.68)	17.90 (16.24, 21.93)	^1^ 0.62 ^2^ 0.09 ^**3**^** 0.04** ^**4**^**0.048**
SBP (mmHg)	118.02 (± 14.60)	110.09 (± 8.33)	115.33 (69.33, 76.67)	114.25 (± 9.51)	^1^ 0.54 ^2^ 0.12 ^3^ 0.64 ^4^0.85
DBP (mmHg)	70.20 (± 10.87)	65.61 (± 8.49)	73.67 (69.33, 76.67)	68.27 (± 8.14)	^1^ 0.62 ^2^ 0.20 ^3^ 0.25 ^4^0.50
WC (cm)	67.65 (57.00, 70.80)	61.97 (± 8.10)	58.40 (55.50, 70.60)	66.54 (± 10.08)	^1^ 0.94 ^2^ 0.13 ^3^ 0.28 ^4^0.65
Common laboratory parameters^†^					
Hb (g/dL)	13.58 (± 1.01)	13.57 (± 0.94)	14.00 (13.40, 14.30)	13.31 (± 1.07)	^1^ 0.73 ^2^ 0.44 ^3^ 0.16 ^4^0.12
Serum creatinine (mg/dL)	0.61 (± 0.08)	0.58 (± 0.11)	0.54 (0.50, 0.61)	0.57 (± 0.11)	^1^ 0.20 ^2^ 0.63 ^3^ 0.82 ^4^0.16
Serum urea (mg/dL)	27.00 (23.00, 29.00)	29.11 (± 5.20)	31.00 (27.00, 37.00)	23.00 (20.00, 28.00)	^1^ 0.13 ^**2**^** 0.007** ^**3**^** 0.011** ^**4**^**0.048**
Serum B2M (mg/L)	1.37 (± 0.21)	1.36 (± 0.24)	1.55 (1.45, 1.67)	1.41 (± 0.23)	^1^ 0.78 ^2^ 0.45 ^3^ 0.054 ^**4**^**0.046**
PTH (pg/mL)	36.60 (27.90, 47.10)	41.80 (23.60, 58.60)	42.10 (29.60, 60.10)	41.90 (31.20, 52.70)	^1^ 0.49 ^2^ 1 ^3^ 0.71 ^4^0.65
Cr-eGFR (mL/min/1.73 m^2^)	103.46 (± 8.00)	100.51 (± 10.73)	99.66 (94.16, 109.29)	105.71 (± 14.87)	^1^ 0.50 ^2^ 0.14 ^3^ 0.59 ^4^0.65
Urinary Ca/Cr	0.11 (± 0.06)	0.07 (0.02, 0.14)	0.10 (0.07, 0.12)	0.06 (0.04, 0.12)	^1^ 0.41 ^2^ 0.97 ^3^ 0.45 ^4^0.77
Albuminuria (uACR ≥ 30 mg/g)	***n* = 17**	***n* = 18**	***n* = 7**	***n* = 34**	
	2 (11.8%)	2 (11.1%)	0 (0.0%)	3 (8.8%)	^1^1 ^2^1 ^3^1 ^4^1
Modern parameters^‡^					
	***n* = 17**	***n* = 19**	***n* = 7**	***n* = 34**	
Serum cystatin C (mg/L)	0.73 (0.49, 0.98)	0.80 (0.61, 0.96)	0.75 (0.64, 0.77)	0.57 (± 0.18)	^1^0.056 ^2^<**0.001** ^3^0.07 ^4^1
Combined Cr-CysC-eGFR (mL/min/1.73 m^2^)	88.29 (84.12, 113.78)	87.24 (79.40, 97.97)	94.26 (82.04, 98.39)	102.86 (96.01, 113.57)	^1^0.08 ^**2**^** < 0.001** ^**3**^** 0.01** ^4^0.46
Serum SDMA (μmol/L)	0.81 (0.72, 0.84)	0.76 (0.66, 0.88)	0.84 (0.81, 0.87)	0.80 (0.73, 0.87)	^1^0.89 ^2^ 0.54 ^3^ 0.37 ^4^0.26

*B2M* β2-microglobulin, *BMI* body mass index, *Ca* calcium, *Cr* creatinine, *CysC* cystatin C, *DBP* diastolic blood pressure, *eGFR* estimated glomerular filtration rate, *ELBW* extremely low birth weight, *Hb* hemoglobin, *LBW* low birth weight, *PTH* parathormone, *SDMA* symmetric dimethylarginine, *SBP* systolic blood pressure, *uACR* urinary albumin to creatine ratio, *VLBW* very low birth weight, *WC* waist circumference

Data are presented as the number of participants, *n*; the number of participants with the percentage in parentheses, *n* (%); the median with interquartile range, median (Q1, Q3); the mean with standard deviation, mean (± SD), as appropriate

^1^LBW vs. controls; ^2^VLBW vs. controls; ^3^ELBW vs. controls; ^4^LBW vs. ELBW

^†^Includes traditional biomarkers (as defined in the text), plus Cr-eGFR

^‡^Includes modern biomarkers (as defined in the text), plus Cr-CysC-eGFR

### Comparison of study groups

Despite initial differences at birth, anthropometric parameters at study inclusion were broadly comparable between the preterm and control groups, except for BMI. Children in the ELBW subgroup had lower BMI compared to controls [16.27 (13.89, 17.68) kg/m^2^ vs. 17.90 (16.24, 21.93) kg/m^2^, *p* = 0.04] (Table 4). Conversely, the LBW subgroup showed a higher mean BMI than term-born children [19.77 (17.38, 21.44) kg/m^2^ vs. 17.90 (16.24, 21.93) kg/m^2^], though the difference was not significant (*p* = 0.62) (Table 4). The combined prevalence of overweight and obesity did not differ between the preterm and control groups [13/43 (30.2%) vs. 15/38 (39.5%); *p* = 0.52], nor did the prevalence of central obesity [3/43 (7.0%) vs. 3/38 (7.9%); *p* = 1.00). Both SBP and DBP were similar between groups [preterm vs. control; SBP: 113.99 (± 11.71) mmHg vs. 114.25 (± 9.51) mmHg, *p* = 0.69; DBP: 68.46 (± 9.48) mmHg vs. 68.27 (± 8.14) mmHg, *p* = 0.93] (Table 3), and likewise, no differences were observed when comparing each preterm subgroup with the controls (Table 4). Notably, the proportion of children with systolic and/or diastolic BP values ≥ 95th percentile was nearly identical between the preterm and reference groups [12/43 (27.9%) vs.11/38 (28.9%), respectively; *p* = 0.92).

Regarding common laboratory parameters, serum urea levels were statistically higher in preterm participants than in controls [27.00 (25.00, 31.00) mg/dL vs. 23.00 (20.00, 28.00) mg/dL, *p* = 0.002] (Table 3), with the difference more evident in the VLBW and ELBW subgroups (Table 4). Additionally, B2M levels were slightly elevated in the ELBW subgroup compared to controls [1.55 (1.45, 1.67) mg/L vs. 1.41 (± 0.23) mg/L, *p* = 0.054], showing a trend toward significance (Table 4). No differences were observed in Hb, serum Cr and Cr-eGFR, PTH, and urinary Ca/Cr values in any comparison (Tables 3 and 4). Importantly, all measurements for the above parameters remained within the age-specific reference range established by our laboratory. Finally, the prevalence of albuminuria (uACR ≥ 30 mg/g) did not differ for the overall preterm group (9.5% vs. 8.8%, *p* = 1.00), or for its subgroups, compared with controls (Tables 3 and 4).

**Fig. 2:**
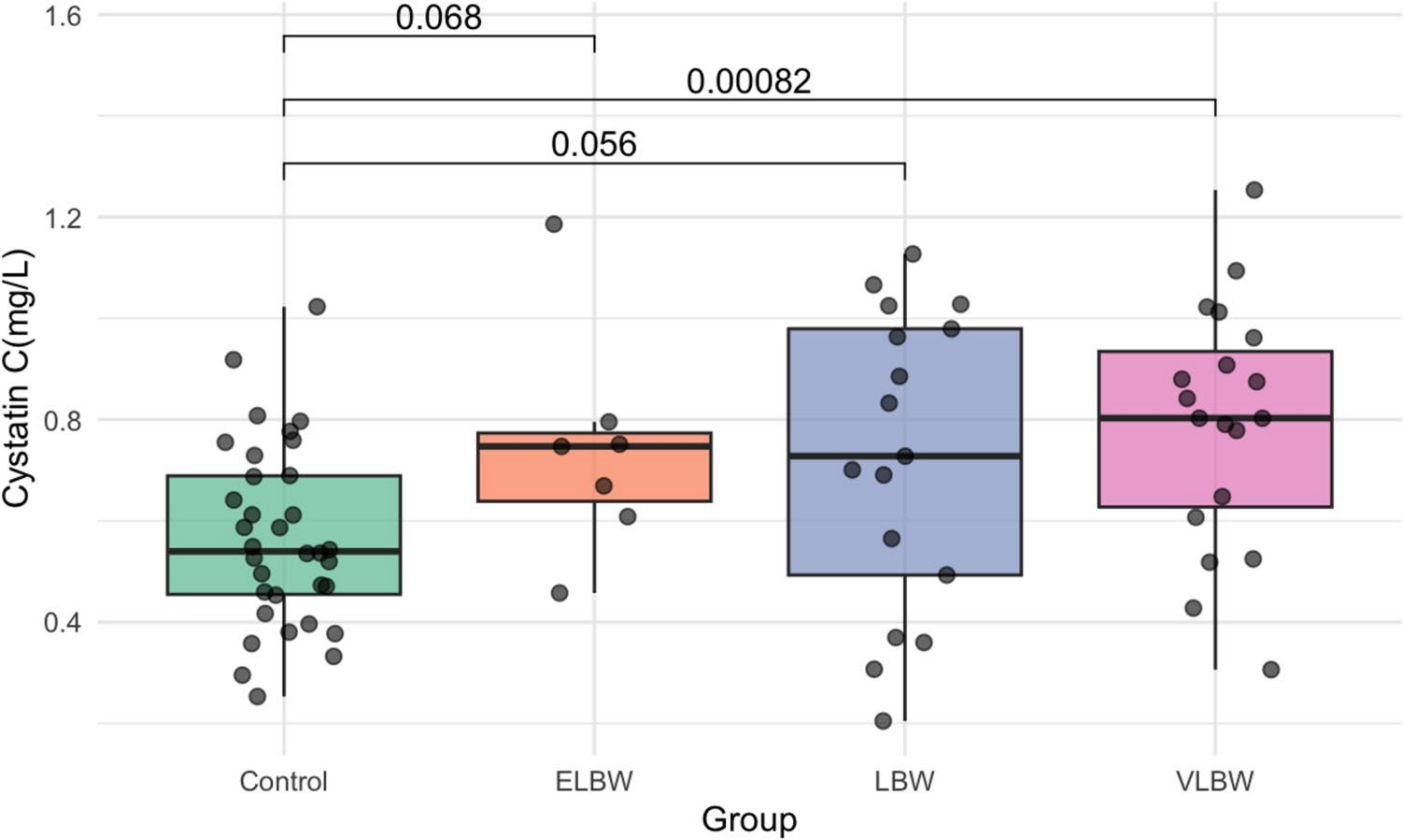
Boxplot analysis of serum cystatin C in preterm subgroups versus controls. *ELBW* extremely low birth weight, *LBW* low birth weight, *VLBW* very low birth weight

**Fig. 3:**
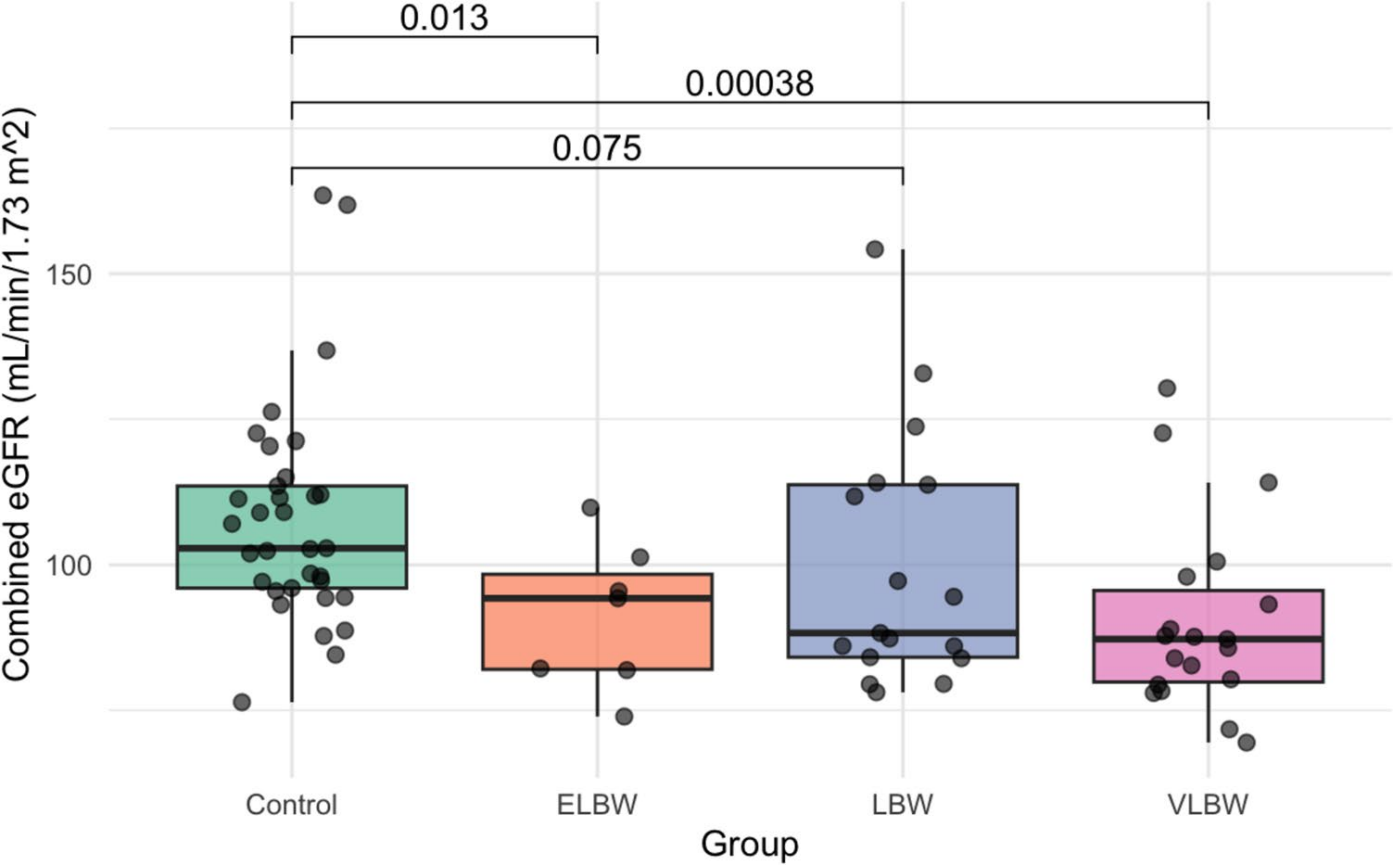
Boxplot analysis of combined Cr-CysC-eGFR in preterm subgroups versus controls. *Cr* creatinine, *CysC* cystatin C, *eGFR* estimated glomerular filtration rate, *ELBW* extremely low birth weight, *LBW* low birth weight, *VLBW* very low birth weigh

Serum cystatin C levels were higher in the premature group [0.76 (± 0.26) mg/L] compared to controls [0.57 (± 0.18) mg/L, *p* < 0.001] (Table 3). Consistently, the combined Cr-CysC-eGFR was lower in preterm children [87.64 (81.90, 101.31) mL/min/1.73m^2^ vs. 102.86 (96.01, 113.57) mL/min/1.73m^2^, *p* < 0.001] (Table 3), indicating reduced kidney function. More specifically, VLBW individuals presented higher median cystatin C levels compared to controls [0.80 (0.61, 0.96) mg/L vs. 0.57 (± 0.18) mg/L, *p* < 0.001] (Table 4), while the ELBW subgroup exhibited a similar trend [0.75 (0.64, 0.77) mg/L vs. 0.57 (± 0.18) mg/L] (Table 4, although it did not reach statistical significance (*p* = 0.07), likely due to small sample size (*n* = 7). These differences in cystatin C and combined Cr-CysC-eGFR values between preterm subgroups and controls are illustrated in Figs. 2 and 3, respectively. In contrast, SDMA levels were similar across all comparisons, with medians of 0.81 (0.68, 0.87) μmol/L in the preterm group and 0.80 (0.73, 0.87) μmol/L in controls (*p* = 0.89) (Tables 3 and 4).

### Comparison of preterm subgroups

When comparing preterm subgroups (Table 4), ELBW participants had lower BMI [16.27 (13.89, 17.68) kg/m^2^ vs. 19.77 (17.38, 21.44) kg/m^2^, *p* = 0.048] and higher B2M levels [1.55 (1.45, 1.67) mg/L vs. 1.37 (± 0.21) mg/L, *p* = 0.046], suggesting potentially greater kidney and metabolic strain in these children. However, albuminuria (uACR ≥ 30 mg/g), observed in 2/17 (11.8%) of LBW and 0/11 (0.0%) of ELBW participants, did not differ between the two subgroups (*p* = 1.00), nor did any of the other assessed markers (e.g., BP, cystatin C, SDMA).

## Discussion

Our study provides further evidence that school-aged children born very preterm, even when apparently healthy and free of major neonatal complications, may already exhibit early subclinical signs of kidney dysfunction. We found higher serum cystatin C levels, along with lower combined Cr-CysC-eGFR in preterm participants compared to term-born peers. These changes emerged despite similar rates of BP ≥ 95th percentile and albuminuria between study groups, highlighting subtle but detectable kidney function impairment at school age and warranting further long-term monitoring.

Large observational studies [30, 31] and meta-analyses [32–34] have consistently demonstrated an inverse association between GA or BW, and long-term kidney health, with individuals born preterm or with LBW exhibiting higher BP and reduced kidney function from childhood into mid-adulthood. This association has been linked to alterations in kidney biomarkers and activation of renin–angiotensin–aldosterone system (RAAS) [1, 3].

Prematurity, LBW, and maternal risk factors (e.g., gestational diabetes, antenatal glucocorticoid exposure, maternal malnutrition) interfere with nephrogenesis, resulting in suboptimal nephron endowment and increased susceptibility to future kidney insults [2, 3]. Although the precise mechanisms remain incompletely understood, the “multi-hit” model suggests that a prenatally programmed nephron deficit renders the kidney more vulnerable to postnatal insults, including impaired perfusion, nephrotoxic agents, obesity, and compensatory hyperfiltration [2, 3, 35].

Despite having fewer nephrons, preterm infants often achieve a glomerular filtration rate (GFR) comparable to term neonates through increased single-nephron workload. However, this adaptive mechanism may predispose to long-term glomerular injury, proteinuria, and hypertension over time [2]. Therefore, preterm and extremely preterm birth confer a two- to three-fold increased risk of CKD, respectively, compared to term-born individuals [31].

In our study, although serum urea was statistically higher in ex-premature individuals compared to controls, particularly in VLBW and ELBW subgroups, all values remained within the normal range, limiting its clinical relevance. Only two comparable studies have examined this outcome, with conflicting results: one reported increased urea in ELBW children [8], while the other found no difference in VLBW adolescents, despite their lower Cr-eGFR compared to term-born controls [36]. Furthermore, these two studies were meta-analyzed by Heo and Lee and yielded non-significant pooled results. Given that serum urea is influenced by multiple non-renal factors, such as dietary protein intake and hydration status, its interpretation as a marker of kidney function should be approached with caution [34].

Although serum B2M levels did not differ between preterm and term-born children overall, a trend toward higher levels was observed specifically in the ELBW subgroup (*p* = 0.054), suggesting a potentially greater kidney burden in this most vulnerable group. To the best of our knowledge, only one similar study—by Staub et al.—has investigated serum Β2M in very preterm children, likewise reporting no significant difference compared to term-born controls [9]. B2M, a low molecular weight protein, freely filtered by the glomerulus and reabsorbed by the proximal tubules, may serve as a dual-function marker of kidney integrity. Given its accessibility and low cost, it could be integrated into multi-marker panels for kidney monitoring in preterm populations, though further validation is warranted [18, 19].

The most salient of our findings was the higher serum cystatin C levels in the preterm group (*p* < 0.001), accompanied by a lower combined Cr-CysC-eGFR (*p* < 0.001), suggesting subtle glomerular dysfunction. Findings from previous studies in similar pediatric populations are inconsistent: roughly half reported elevated cystatin C in preterm-born children [7, 8, 11], while the others found no significant differences compared to term-born controls [9, 10, 13]. Such heterogeneity likely reflects differences in age at assessment, sample size, degree of prematurity, and analytical methods. Notably, Heo and Lee’s meta-analysis also found no significant difference in cystatin C levels between preterm and term-born individuals, but it included only three studies—one of which involved adults—limiting its applicability to pediatric cohorts [34]. Given its independence from muscle mass, age, and sex, cystatin C is considered a representative marker of early kidney dysfunction, particularly in preterm populations. It can detect impairment before serum creatinine rises, and eGFR equations incorporating cystatin C (with or without Cr), have demonstrated superior accuracy in children [16].

Regarding SDMA, only two previous studies have investigated its levels in preterm or LBW children, both reporting higher concentrations than in controls [10, 22]. In contrast, our analysis did not reveal any significant differences across group comparisons. This low-molecular-weight arginine derivative, excreted almost exclusively by the kidneys, is muscle-mass-independent and shows stable levels across the pediatric age range. We included it in our panel as a modern biomarker to complement more frequently used indicators of early kidney dysfunction [21, 22]. Its lack of elevation in our cohort may reflect sample characteristics, limited statistical power, or the possibility that SDMA increases only later in life or in individuals with more advanced subclinical kidney impairment.

No significant difference in the frequency of albuminuria (uACR ≥ 30 mg/g) was observed between preterm and term-born children. Although albuminuria is a recognized marker of glomerular hyperfiltration, this finding may reflect preserved kidney function in our clinically stable cohort, free from major neonatal complications. Similar findings have been reported in previous pediatric studies [7, 13, 36, 37], whereas higher uACR levels have been observed in young adults born very preterm compared to full-term controls, suggesting that glomerular injury and albuminuria may evolve gradually and become more evident with age [38].

Consistent with most comparable studies [10, 12, 13, 39], we observed no significant differences in SBP or DBP between preterm and full-term groups. A few studies, however, have reported higher BP in preterm cohorts. In some, these findings emerged only under specific methodological conditions, such as sex-stratified analysis [9], use of BP *z*-scores [40], or supine rather than seated measurements [41]. In others [42, 43], the reasons for the observed differences remain unclear, but may relate to sample variability or unmeasured confounders. Interestingly, we found a relatively high proportion of children with BP values ≥ 95th percentile in both groups, particularly among controls (preterm: 27.9%; full-term: 28.9%). This may reflect transient anxiety related to the upcoming blood draw or a white coat effect.

Although BP and albuminuria did not differ between groups, the lower Cr-CysC-eGFR in preterm participants could reflect subclinical nephron alterations. Early tubular injury may also contribute to this dysfunction. Supporting this, Hingorani et al. found that higher urinary α-glutathione-S-transferase (αGST) levels—a biomarker of proximal tubular cell injury—during NICU stay were associated with reduced Cr–CysC eGFR at two years of age in extremely preterm infants [44]. Since all individual values of Cr-based eGFR remained normal (> 90 mL/min/1.73 m^2^), this finding likely represents a subtle decline in functional nephron mass rather than overt dysfunction. Such early changes, detectable only through more sensitive markers like cystatin C, may precede hypertension or proteinuria, in line with the multistep progression described by the glomerular hyperfiltration hypothesis [2, 3].

Like Vollsæter et al., but in contrast to Yamamura-Miyazaki et al., we observed no overall difference in BMI between preterm and full-term children [10, 11]. However, BMI was lower in the ELBW subgroup compared to controls (*p* = 0.04). Within the preterm group, ELBW children also showed lower BMI (*p* = 0.048), as well as higher B2M levels (*p* = 0.046), than their LBW counterparts. These findings suggest that even among children born at similar GA, lower BW may be associated with subtle long-term alterations in growth and kidney parameters. However, they should be interpreted with caution due to the small sample size of the ELBW subgroup (*n* = 7).

The use of NSAIDs for pharmacologic PDA closure during the early neonatal period may impair ongoing nephrogenesis (which continues for approximately 40 days postnatally in preterm infants) by inhibiting prostaglandins and reducing renal perfusion [45]. In our study, the two ELBW monozygotic twin girls who developed nephrocalcinosis had both been exposed to NSAIDs and prolonged diuretic therapy, raising the possibility of a cumulative nephrotoxic effect in the setting of shared genetic susceptibility. Follow-up ultrasounds 3 months post-discharge showed complete resolution, in line with previously reported favorable outcomes [46].

Our study has several strengths and limitations. Strengths include the use of an age- and sex-matched control group and a multi-marker approach, combining traditional and modern biomarkers. The upper limit of 32 weeks gestation was chosen to reflect disrupted nephrogenesis [4]. Moreover, the sample size, although modest, was comparable to those used in similar exploratory studies in pediatric nephrology.

The preterm group comprised apparently healthy school-aged children, without a history of SGA, AKI, or major neonatal complications, and with low incidence of prolonged mechanical ventilation. While not representative of more complex preterm survivors, this cohort represents a common real-world outpatient group often perceived as low risk, whose potential subclinical kidney vulnerability we aimed to investigate. The absence of AKI cases likely reflects its clinical profile (AKI was not an exclusion criterion). Regardless, recent evidence suggests no association between neonatal AKI and adverse kidney outcomes in adolescence [47].

A further consideration concerns the high proportion of participants from multiple pregnancies (twins and triplets). While this pattern aligns with current trends in very preterm births, driven by increased use of assisted reproductive technologies, it may limit the generalizability of our findings to singleton populations. Twin gestations carry a higher risk for long-term cardio-renal complications, particularly when associated with FGR, preterm delivery, or TTTS [48]. However, in the absence of severe FGR—as was the case in our cohort—kidney development and function in twins appear to be comparable to those in singletons [49, 50]. Finally, our analysis did not include imaging data.

It is well established that nephrons do not regenerate, making the preservation of functional nephron mass for as long as possible crucial in individuals with reduced kidney endowment. While no specific therapy can halt the progression of chronic kidney dysfunction, early identification of vulnerable individuals and implementation of nephroprotective strategies are of paramount importance [1].

## Conclusion

In this study, children born very preterm demonstrated significantly higher cystatin C levels and lower combined Cr-CysC-eGFR values compared to full-term peers, suggesting early kidney dysfunction. Among preterm subgroups, ELBW children appeared particularly vulnerable, exhibiting lower BMI and higher B2M levels than their LBW counterparts. These findings support the use of cystatin C and combined Cr-CysC-eGFR as sensitive biomarkers for early detection of kidney impairment in this high-risk population. Further longitudinal studies are warranted to validate the diagnostic utility of cystatin C and other promising biomarkers and guide optimized long-term kidney surveillance strategies for survivors of very preterm birth.

## Supplementary Information

Below is the link to the electronic supplementary material.

Graphical abstract (PPTX 160 KB)

## Data Availability

The datasets generated during and/or analyzed during the current study are available from the corresponding author on reasonable request.
