# Supplementary material for: Early detection of kidney impairment in school-aged children born very preterm: a parallel use of traditional and modern biomarkers
Source: Pediatr Nephrol. 2025 Jul 7;41(2):423–36. doi: 10.1007/s00467-025-06876-1 (PMC12727860; doi:10.1007/s00467-025-06876-1)
Supplement: Supplementary file 1 — Graphical abstract (PPTX 160 KB) [file 467_2025_6876_MOESM1_ESM.pptx]

## Slide 1
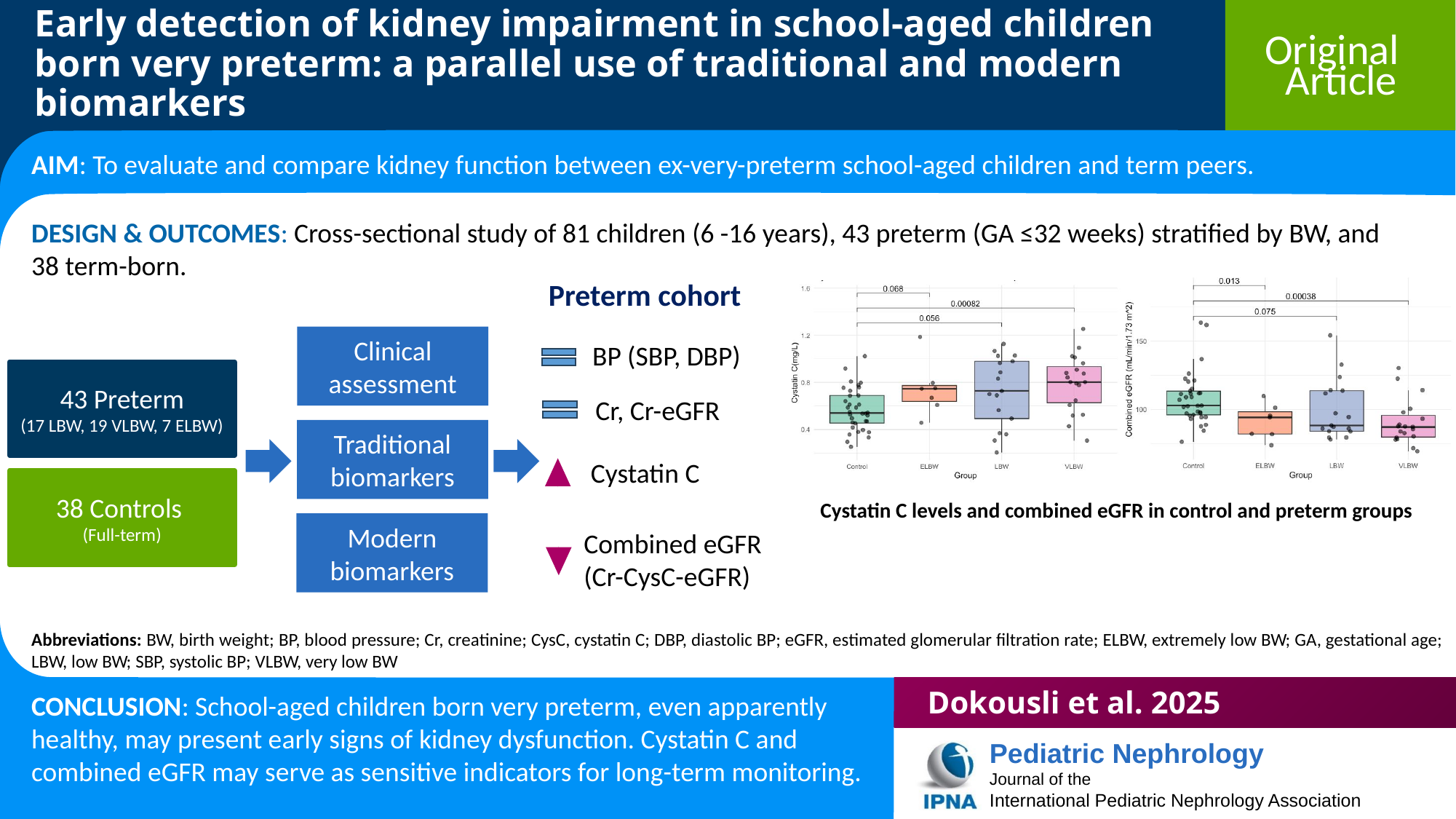

Early detection of kidney impairment in school-aged children born very preterm: a parallel use of traditional and modern biomarkers
AIM: To evaluate and compare kidney function between ex-very-preterm school-aged children and term peers.
DESIGN & OUTCOMES: Cross-sectional study of 81 children (6 -16 years), 43 preterm (GA ≤32 weeks) stratified by BW, and 38 term-born.
Preterm cohort
Clinical assessment
BP (SBP, DBP)
43 Preterm
(17 LBW, 19 VLBW, 7 ELBW)
Cr, Cr-eGFR
Traditional biomarkers
Cystatin C
38 Controls
(Full-term)
Cystatin C levels and combined eGFR in control and preterm groups
Modern biomarkers
Combined eGFR
(Cr-CysC-eGFR)
Abbreviations: BW, birth weight; BP, blood pressure; Cr, creatinine; CysC, cystatin C; DBP, diastolic BP; eGFR, estimated glomerular filtration rate; ELBW, extremely low BW; GA, gestational age; LBW, low BW; SBP, systolic BP; VLBW, very low BW
Dokousli et al. 2025
CONCLUSION: School-aged children born very preterm, even apparently healthy, may present early signs of kidney dysfunction. Cystatin C and combined eGFR may serve as sensitive indicators for long-term monitoring.
